# Sofosbuvir protects Zika virus-infected mice from mortality, preventing short- and long-term sequelae

**DOI:** 10.1038/s41598-017-09797-8

**Published:** 2017-08-25

**Authors:** André C. Ferreira, Camila Zaverucha-do-Valle, Patrícia A. Reis, Giselle Barbosa-Lima, Yasmine Rangel Vieira, Mayara Mattos, Priscila de Paiva Silva, Carolina Sacramento, Hugo C. de Castro Faria Neto, Loraine Campanati, Amilcar Tanuri, Karin Brüning, Fernando A. Bozza, Patrícia T. Bozza, Thiago Moreno L. Souza

**Affiliations:** 1 Laboratório de Imunofarmacologia, Instituto Oswaldo Cruz (IOC), Fundação Oswaldo Cruz (Fiocruz), Rio de Janeiro, RJ Brazil; 2 Instituto Nacional de Infectologia (INI), Fiocruz, Rio de Janeiro, RJ Brazil; 3 Instituto de Ciências Biomédicas, Universidade Federal do Rio de Janeiro, Rio de Janeiro, RJ Brazil; 4 Instituto de Biologia, Universidade Federal do Rio de Janeiro (UFRJ), Rio de Janeiro, RJ Brazil; 5 BMK Consortium: Blanver Farmoquímica Ltda; Microbiológica Química e Farmacêutica Ltda, Karin Bruning & Cia. Ltda, Taboão da Serra, SP Brazil; 6 National Institute for Science and Technology on Innovation on Neglected Diseases (INCT/IDN), Center for Technological Development in Health (CDTS), Fiocruz, Rio de Janeiro, RJ Brazil

## Abstract

Zika virus (ZIKV) causes significant public health concerns because of its association with congenital malformations, neurological disorders in adults, and, more recently, death. Considering the necessity to mitigate ZIKV-associated diseases, antiviral interventions are an urgent necessity. Sofosbuvir, a drug in clinical use against hepatitis C virus (HCV), is among the FDA-approved substances endowed with anti-ZIKV activity. In this work, we further investigated the *in vivo* activity of sofosbuvir against ZIKV. Neonatal Swiss mice were infected with ZIKV (2 × 10^7^ PFU) and treated with sofosbuvir at 20 mg/kg/day, a concentration compatible with pre-clinical development of this drug. We found that sofosbuvir reduced acute levels of ZIKV from 60 to 90% in different anatomical compartments, such as the blood plasma, spleen, kidney, and brain. Early treatment with sofosbuvir doubled the percentage and time of survival of ZIKV-infected animals. Sofosbuvir also prevented the acute neuromotor impairment triggered by ZIKV. In the long-term behavioural analysis of ZIKV-associated sequelae, sofosbuvir prevented loss of hippocampal- and amygdala-dependent memory. Our results indicate that sofosbuvir inhibits ZIKV replication *in vivo*, which is consistent with the prospective necessity of antiviral drugs to treat ZIKV-infected individuals.

## Introduction

Zika virus (ZIKV) is an enveloped, positive-sense, single stranded RNA pathogen that belongs to the *Flaviviridae* family. ZIKV is transmitted by mosquitoes, similar to several other arboviruses of the *Flavivirus* genus, such as dengue virus (DENV). ZIKV re-emerged in the last few years and was revealed to be a unique pathogen^1^. ZIKV is spread through sexual and physical contact, as well as vertically^1^. The main wave of Zika epidemics in the Americas occurred from the middle of 2015 to the beginning of 2016, when the World Health Organization (WHO) declared this outbreak as a public health emergency of international concern. This relevant apprehension was raised due to ZIKV’s association with congenital malformations, including microcephaly, and a broad range of neurological disorders in adults, including Guillain-Barré syndrome^2, 3^. With the number of cases rising, Zika-associated deaths have also been reported^4, 5^. Considering the necessity to mitigate ZIKV-associated morbidities, antiviral interventions against this virus are an urgent necessity.

Different studies have been published on the repositioning of Food and Drug Administration (FDA)-approved molecules to treat ZIKV infection^6–11^. Over 30 FDA-approved molecules are endowed with anti-ZIKV activity. Among them, we and others have shown that sofosbuvir, a clinically approved anti-hepatitis C virus (HCV), targets ZIKV RNA polymerase, leading to inhibition of virus replication in cellular types important for the pathogenesis of this emergent agent, such as human brain organoids, neural stem cells, and neuroepithelial stem cells^12–14^. Therefore, sofosbuvir may represent a selective option to treat Zika. Nevertheless, more detailed analyses of sofosbuvir’s anti-ZIKV activity *in vivo* are necessary.

Different animal models of Zika virus infection have been reported recently^15^. Many of these models, from immunocompromised to immunocompetent neonatal mice, among others, are relevant for antiviral testing^15^. In general, for pharmacological studies, outbred animals, such as Swiss mice, represent a consistent model. These animals display broader responses often found in heterogeneous populations^16^, such as humans. Indeed, ZIKV infection in Swiss neonatal mouse models has been characterized since 1950^15, 17–19^. Another advantage of neonatal mouse models is the opportunity to further examine behavioural sequelae induced by the infection and whether treatments could overcome/prevent this phenotype.

In this study, we show that sofosbuvir protects ZIKV-infected animals from mortality by a very significant viral challenge. This was associated with a decrease in viral RNA levels in different tissues and prevention of acute neuromotor and long-term memory sequelae.

## Material and Methods

### Reagents

The antiviral drug sofosbuvir (β-D-2′-deoxy-2′-α-fluoro-2′-β-C-methyluridine) was donated by the BMK Consortium: Blanver Farmoquímica Ltda; Microbiológica Química e Farmacêutica Ltda; Karin Bruning & Cia. Ltda, (Taboão da Serra, São Paulo, Brazil). Drugs were dissolved in 100% dimethylsulfoxide (DMSO) 1:10 (mass/volume), followed by the appropriate dilutions in PBS or culture medium (DMEM) to treat the animals. The final DMSO concentrations showed no toxicity to the animals. Other materials were purchased from Thermo Scientific Life Sciences (Grand Island, NY) unless otherwise mentioned.

### Cells

African green monkey kidney (Vero) cells were cultured in DMEM. The culture medium was supplemented with 10% foetal bovine serum (FBS; HyClone, Logan, Utah), 100 U/mL penicillin, and 100 µg/mL streptomycin. Cells were kept at 37 °C in 5% CO_2_.

### Virus

ZIKV African (MR766) and Brazilian (GenBank accession #KX19720513) strains were propagated in Vero cells (E6 subtype). Vero cells were infected at a multiplicity of infection (MOI) of 0.01 at 37 °C for 1 h. Next, unadsorbed virus particles were removed by washing with phosphate-buffered saline (PBS), and the cells were cultured at 70% confluency for an additional 5 to 7 days in medium with 1% FBS. After each period, the cells were lysed by freezing and thawing and centrifuged at 1,500 × *g* at 4 °C for 20 min to remove cellular debris. The virus was stored at −70 °C for further studies. Detailed protocols to grow the stock virus strains at titers ~10^9^ PFU/mL have been described by us previously^13, 20^.

### Plaque-forming assay

Monolayers of Vero cells in 6-well plates were exposed to different dilutions of the supernatant containing virus for 1 h at 37 °C. Next, the cells were washed with PBS, and DMEM containing 1% FBS and 3% carboxymethylcellulose (Fluka) (overlay medium) was added to the cells. After 5 days at 37 °C, the monolayers were fixed with 10% formaldehyde in PBS and stained with a 0.1% solution of crystal violet in 70% methanol. The virus titers were then calculated by scoring the plaque-forming units (PFU).

### Molecular detection of virus RNA levels

Total RNA from culture, extract-containing organs in PBS, or plasma was extracted using QIAamp Viral RNA or RNeasy Mini Kits (Qiagen®), according to manufacturer’s instructions. Quantitative RT-PCR was performed using QuantiTect or QuantiNova Probe RT-PCR Kit (Quiagen®) in an ABI PRISM 7300 Sequence Detection System (Applied Biosystems). Amplifications were carried out in 25 µL reaction mixtures containing 2 × reaction mix buffer, 50 µM of each primer, 10 µM of probe, and 5 µL of RNA template. Primers, probes, and cycling conditions recommended by the Centers for Disease Control and Prevention (CDC) protocol were used to detect the ZIKV^21^. The standard curve method was employed for virus quantification. For reference on the cell amounts used, the housekeeping gene RNAse P was amplified^20^. The Ct values for this target were compared to those obtained to different cell amounts, 10^7^ to 10^2^, for calibration.

### Animals

Swiss albino mice (*Mus musculus*) (pathogen-free) from the Oswaldo Cruz Foundation breeding unit (Instituto de Ciência e Tecnologia em Biomodelos (ICTB)/Fiocruz) were used for these studies. The animals were kept at a constant temperature (25 °C) with free access to chow and water in a 12-h light/dark cycle. The experimental laboratory received pregnant mice (at approximately the 14th gestational day) from the breeding unit. Pregnant mice were observed daily until delivery to accurately determine the postnatal day. We established a litter size of 10 animals for all experimental replicates.

The Animal Welfare Committee of the Oswaldo Cruz Foundation (CEUA/FIOCRUZ) approved and covered (license number L-016/2016) the experiments in this study. The procedures described in this study were in accordance with the local guidelines and guidelines published in the National Institutes of Health Guide for the Care and Use of Laboratory Animals. The study is reported in accordance with the ARRIVE guidelines for reporting experiments involving animals^22^.

### Experimental infection and treatment

Three-day-old Swiss mice were infected intraperitoneally with 2 × 10^7^ PFU of virus^18, 23^, unless otherwise mentioned. Treatments with sofosbuvir were carried out with sofosbuvir at 20 mg/kg/day intraperitoneally. Treatment started one day prior to infection (pretreatment) or two days after infection (late treatment). In both cases, treatment was conducted for 7 days. For comparisons, mock-infected and mock-treated groups of animals were used as controls. Animals were monitored daily for survival, weight gain, and virus-induced short-term sequelae (righting in up to 60 seconds) (Supplementary Video).

Blood was collected by cardiac puncture and placed on citrate-containing tubes for plasma separation. Tissues (spleen, kidney, and brain) were collected. Initially, the tissues were analysed macroscopically for the presence of pathological signs. Whenever possible, the pathological signs were quantified by counting per verified tissue/organs. Alternatively, tissues were lysed (RLT buffer; Qiagen) and homogenized with a Potter-Elvehjem homogenizer (Teflon pestle and glass mortar). Homogenates were cleared by centrifugation, and total RNA was extracted.

If necessary to alleviate animal suffering, euthanasia was performed. The criteria were the following: i) differences in weight gain between infected and control groups > 50%, ii) ataxia, iii) loss of gait reflex, iv) absence of righting reflex within 60 seconds, and v) separation, with no feeding, of moribund offspring by the female adult mouse.

### Behavioural tests

To test the righting reflex, animals were tested daily during the course of acute infection. Animals were held in a supine position with all four paws facing up in the air for 5 seconds. Then, animals were released, and the time the animal took to flip over onto its stomach with all four paws touching the surface was measured. A maximum of 60 seconds was given for each trial, and animals were tested twice a day with a 5-minute minimum interval between trials. For each animal, the lowest time was plotted in the graph. Animals that failed the test were included in the graph with a time of 60 seconds. Please see the Supplementary Video.

Animals at 6 to 8 weeks of age were assayed for long-term sequelae by different tests. The Morris water maze (MWM) is a behavioural task to evaluate hippocampal-dependent learning and spatial memory. The water maze comprised a black circular pool (100 cm in diameter) that was conceptually divided into four equal, but imaginary, quadrants for the purpose of data analysis. The water temperature was 25 °C. A platform (10 cm^2^), which was hidden from the mouse’s view, was located 2 cm beneath the surface of the water, allowing the mouse to easily climb onto it once its presence was detected. The water maze was located in a well-lit white room with several posters and other distal visual stimuli hanging on the walls to provide spatial cues. Training on the hidden platform (spatial) version of the MWM was carried out on 4 consecutive days. On day 5, when the platform was removed, memory was evaluated.

An amygdala-dependent aversive memory assay (freezing test) was conducted in a chamber with 3 dark walls and clear frontal wall and lid (28 × 26 × 23 cm). The floor of the chamber consisted of 20 parallel stainless steel grid bars, each measuring 4 mm in diameter and spaced 7 cm apart. The grid was connected to a shock generator device (Insight LTDA, Brazil). A training session consisted of placing the mouse in the chamber and allowing a 3-min acclimation period. After, mice received two foot shocks (0.6 mA, 3 s with one interval of 30 s) and were then returned to their home cages. Twenty-four hours later, mice were exposed to the same environment without shock stimuli for 3 min. Memory was assessed and expressed as the percentage of time that mice spent freezing (considered crouching without movement of the head except when associated with breathing).

### Statistical analysis

Significance of survival curves was evaluated using the Log-rank (Mantel-Cox) test. Behavioural tests were analysed with ANOVA, followed by Tukey’s post hoc test using Graphpad Prism software 7.0. Odds ratios (OR) and 95% confidence intervals (CI) were calculated by Fisher’s exact test with Lancaster’s mid-P correction using OpenEpi software^24^ for comparisons of mortality among groups. *P* values of 0.05 or less were considered statistically significant.

## Results

### Sofosbuvir enhances the survival of ZIKV-infected mice

To evaluate whether sofosbuvir, at a concentration compatible with the pre-clinical/clinical studies for drug approval^25^, protects ZIKV-infected mice, we first determined the strain and dose of virus to be used. Upon intraperitoneal infection, we found that the African strain induced greater mortality than the Brazilian virus (Figure S1A), in line with previous studies^19, 26^. Subsequently, we determined that the dose of 2 × 10^7^ PFU of African ZIKV was severe enough to drive mouse mortality within a week or less (Figure S1B). The next experiments were conducted using this condition.

**Figure 1:**
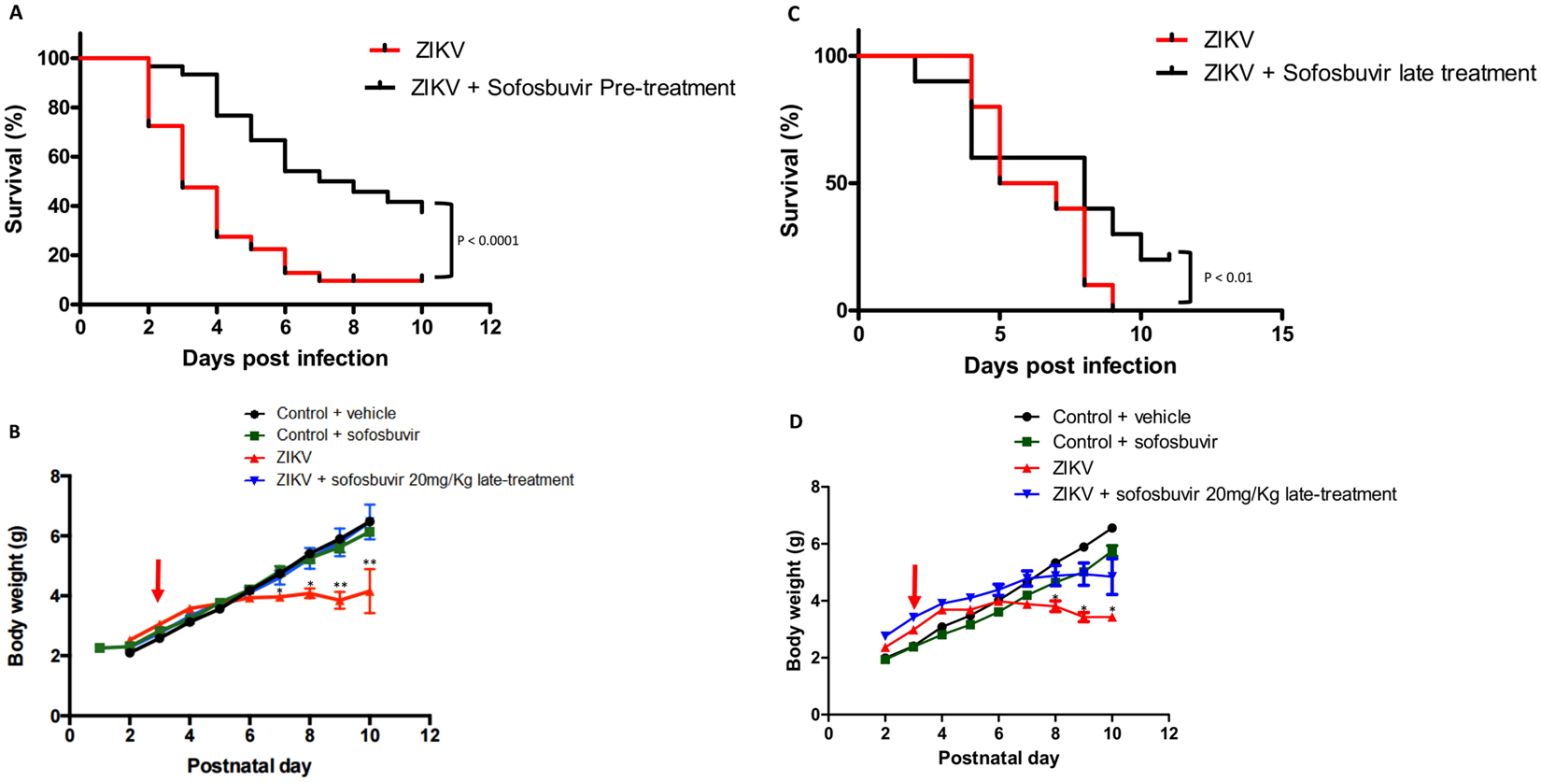
Treatment with Sofosbuvir increases survival and inhibits weight loss of ZIKV-infected mice. Three**-**day-old Swiss mice were infected with ZIKV (2 × 10^7^ PFU) and treated with sofosbuvir either 1 day before (**A** and **B**) or 2 days after infection (**C** and **D**). Survival (**A** and **C**) and weight variation (**B** and **D**) were assessed during the course of treatment (7 days). The red arrow indicates when animals were infected. Survival was statistically assessed by Log-rank (Mentel-Cox) test. Differences in weight are displayed as the means ± SEM, and two-way ANOVA for each day was used to assess the significance. Independent experiments were performed with at least 10 mice/group (n = 50). *P < 0.01; **P < 0.001.

Sofosbuvir significantly protected pretreated infected mice from ZIKV-induced mortality (Fig. 1A). Virtually all ZIKV-infected mice died up to 7 days after infection, whereas 40% of sofosbuvir-treated ZIKV-infected animals survived (Fig. 1A). Along with the survival curves, we evaluated the weight gain of pretreated animals during the time course of the assay. ZIKV-infected animals had impaired postnatal development, whereas the weight gain of sofosbuvir-treated ZIKV-infected mice was indistinguishable from uninfected controls (Fig. 1B).

Postponing the treatment to the second day after infection still preserved some level of protection to ZIKV-infected mice (Fig. 1C). Untreated ZIKV-infected mice died at 8 days post-infection, whereas 25% of the mice receiving late treatment with sofosbuvir survived (Fig. 1C). With respect to the postnatal development of ZIKV-infected mice, treated animals gained more weight than untreated mice (Fig. 1D).

**Figure 2:**
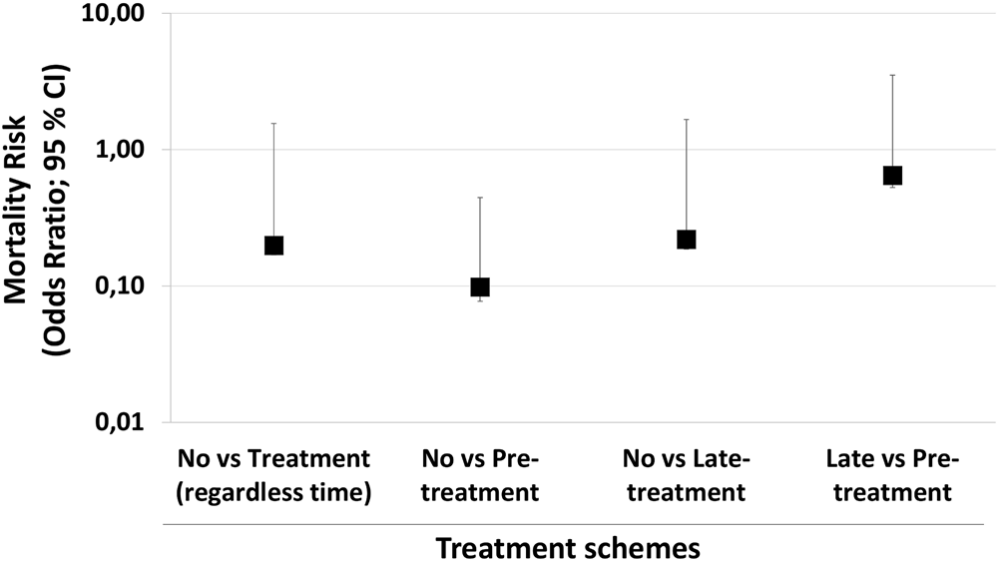
Sofosbuvir reduces the risk mortality of ZIKV-infected mice. Taking the data from Fig. 1A,C as reference, the odds ratio for mortality risk (with 95% confidence interval; CI) was calculated by Fisher’s exact test with Lancaster’s mid-P correction. All comparisons were statistically significant, with P < 0.001.

Remarkably, treatment regimens resulted in enhanced survival when compared to the absence of treatment. Nevertheless, pretreatment may be considered more effective. Pretreatment decreased the mortality rates and increased the mean time of survival (T_50_) when compared to late treatments (Table 1). To statistically compare different experimental groups with respect to mortality risk, odds ratios (OR) for this outcome were calculated. Comparing no treatment with sofosbuvir-treated mice (irrespective of timing) had a reduced mortality risk (OR = 0.198) (Fig. 2). However, compared with no treatment, pretreatment was more effective than the late regimen (no vs. pre: OR = 0.098; no vs. late OR = 0.220) (Fig. 2). The comparison between late vs pretreatment also revealed the benefits of earlier interventions (OR = 0.648) (Fig. 2). Our results show that sofosbuvir treatment is associated with reduced mortality in infected mice. Although potential benefits from sofosbuvir treatment were observed regardless of when animals received the intervention, earlier administration resulted in substantially improved antiviral results.

**Table 1:** Percentage of mortality and mean survival time of sofosbuvir-treated ZIKV-infected mice.

	Treatment Schemes			
	Pre		Late	
	Nil	Sofosbuvir	Nil	Sofosbuvir
Mortality (%)	95	40	100	25
T50 (Days)	3	7	5	7

### Sofosbuvir decreases ZIKV loads during acute infection

**Figure 3:**
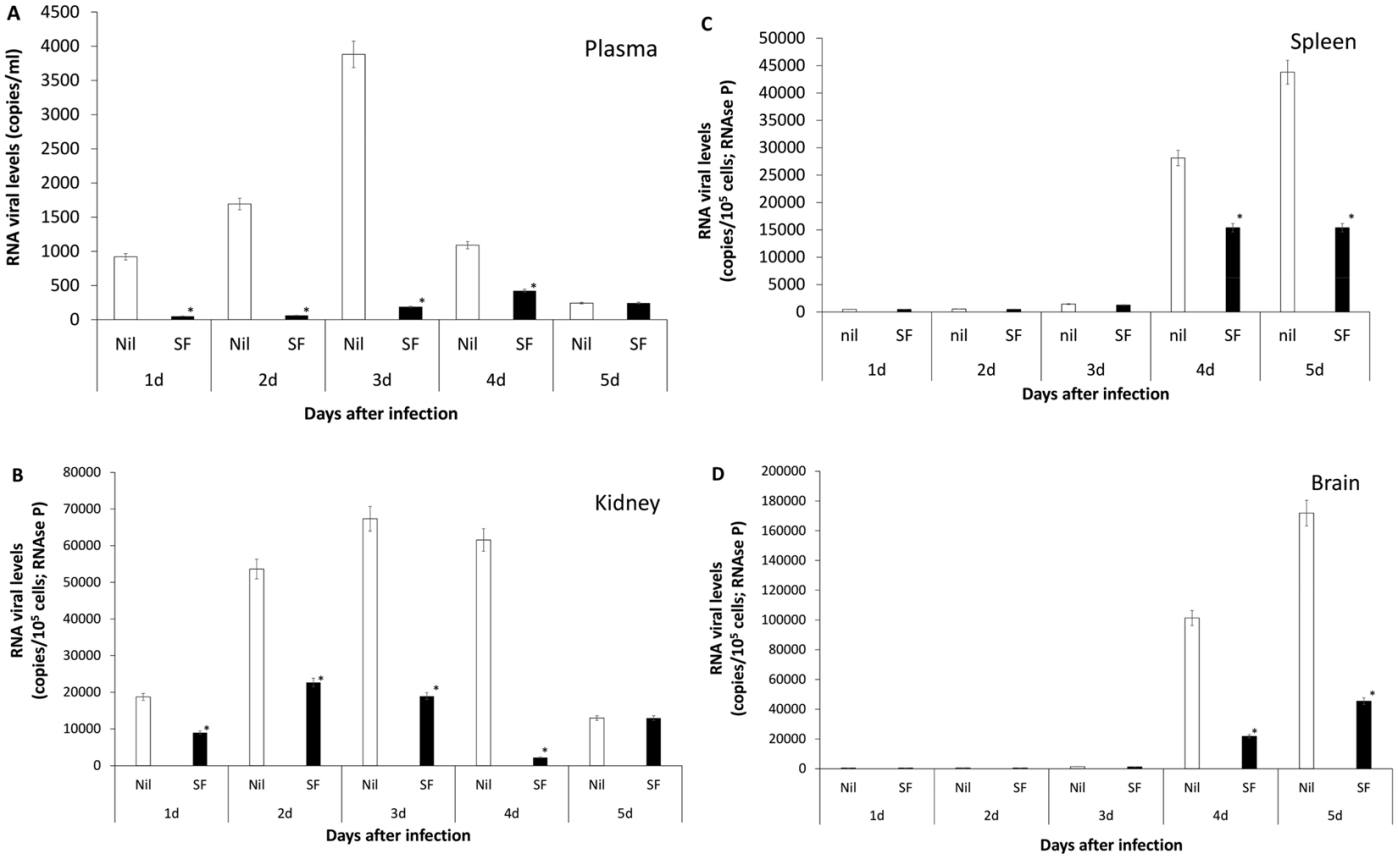
Sofosbuvir-dependent inhibition of ZIKV replication reduces viral loads in different anatomical compartments during acute infection. Three**-**day-old Swiss mice were infected with ZIKV (2 × 10^7^ PFU) and treated with sofosbuvir (SF) or not (nil) beginning 1 day before infection. At indicated days after infection, animals were euthanized, and ZIKV RNA levels were measured in the plasma (**A**), kidney (**B**), spleen (**C**), and brain (**D**). The results are displayed as the means ± SEM (at least three technical replicates from at least three mice per group per day were assayed). Student’s t test was used to compare the viral levels from SF- vs. mock-treated mice. *P < 0.01.

**Figure 4:**
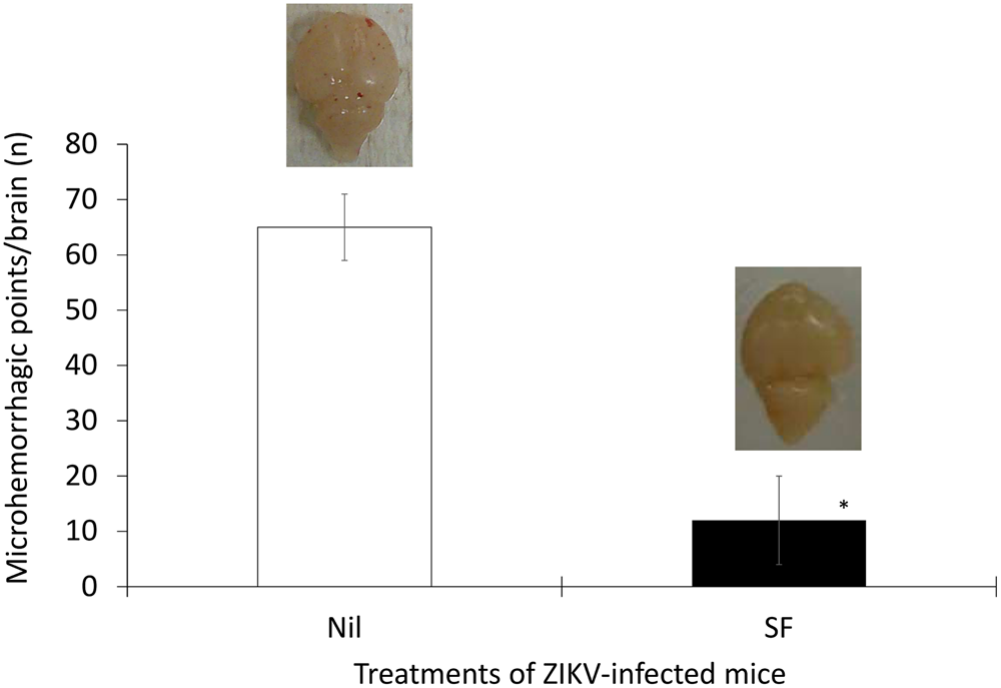
Sofosbuvir decreases the degree of microhaemorrhage in the brains of ZIKV-infected mice. Three**-**day-old Swiss mice were infected with ZIKV (2 × 10^7^ PFU) and treated with sofosbuvir (SF) or not (nil) beginning 1 day before infection. At the fifth day after infection, animals were euthanized and whole brain collected to quantify the microhaemorrhagic foci. The means ± SEM of at least three mice per group are displayed. Student’s t test was used to compare the viral levels of SF- vs. mock-treated mice. *P < 0.01. The insets are representative brains of untreated and treated mice.

Since sofosbuvir inhibits ZIKV replication, enhanced survival due to this treatment was presumably associated with reduction in viral levels during acute infection. We evaluated this hypothesis and measured the magnitude of virus inhibition *in vivo*. To do so, sofosbuvir-treated ZIKV-infected animals were euthanized daily from the first to the fifth day after infection. Next, viral loads were measured in different tissues (Fig. 3). We observed that sofosbuvir reduced the mice viraemia by over 90%, especially during the first 3 days after infection, when an exponential increase in viral levels in the plasma was observed (Fig. 3A). Sofosbuvir reduced the peak of virus detection by up to 60% between 2 and 4 days post-infection in the kidney (Fig. 3B). Virus detection in the spleen and brain was abundant at 4 to 5 days after infection, and sofosbuvir treatment reduced virus levels in these tissues by up to 80% (Fig. 3C,D). Remarkably, during this experiment, we observed that ZIKV-infected animals had foci of cerebral microhaemorrhage. Sofosbuvir prevented/reduced ZIKV-induced microhaemorrhage by over 90% (Fig. 4) in pretreated animals.

Altogether, our results indicate that the effects of sofosbuvir on mouse survival were indeed followed by a reduction in virus detection in different anatomical compartments.

### Sofosbuvir prevents short- and long-term sequelae in ZIKV-infected mice

**Figure 5:**
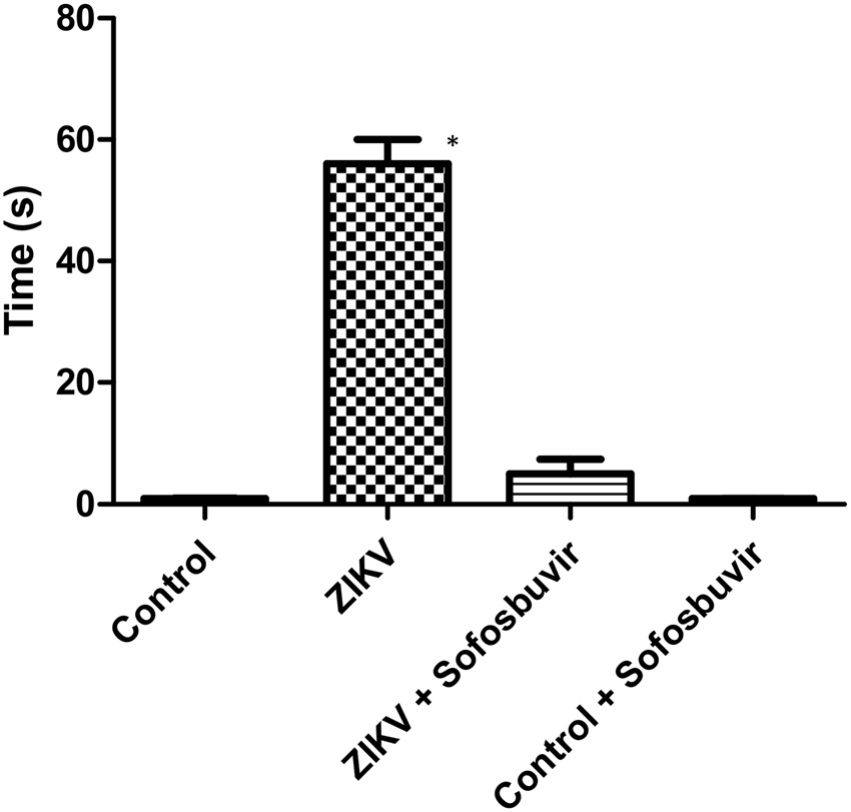
Sofosbuvir prevents neuromotor impairment in ZIKV-infected mice. Three**-**day-old Swiss mice were infected with ZIKV (2 × 10^7^ PFU) and treated with sofosbuvir (SF) or not (nil) beginning 1 day before infection. At the fifth day after infection, animals were tested for righting (Supplementary Video). Animals were turned backwards and allowed up to 60 s to return to the upright position. The results are presented as the means ± SEM. This was a routine measure and at least 10 animals per group were analysed. Student’s t test was used to compare untreated ZIKV-infected mice with other groups individually. *P < 0.01.

The neonatal animal model may represent a relevant model to evaluate short, and especially, long-term behavioural sequelae after infection. Consistently, we observed an acute neuromotor impairment in ZIKV-infected mice (Supplementary Video). To determine the magnitude of this injury and the benefits of sofosbuvir use, we applied the righting test reflex for up to 60 seconds. ZIKV-infected animals, untreated with sofosbuvir, took 12 times longer to stay in the upright position than sofosbuvir-treated animals or controls (Fig. 5). Sofosbuvir-treated ZIKV-infected mice and the controls were statistically indistinguishable (Fig. 5). Our data indicate that sofosbuvir protects animals from ZIKV-associated acute neuromotor impairment.

**Figure 6:**
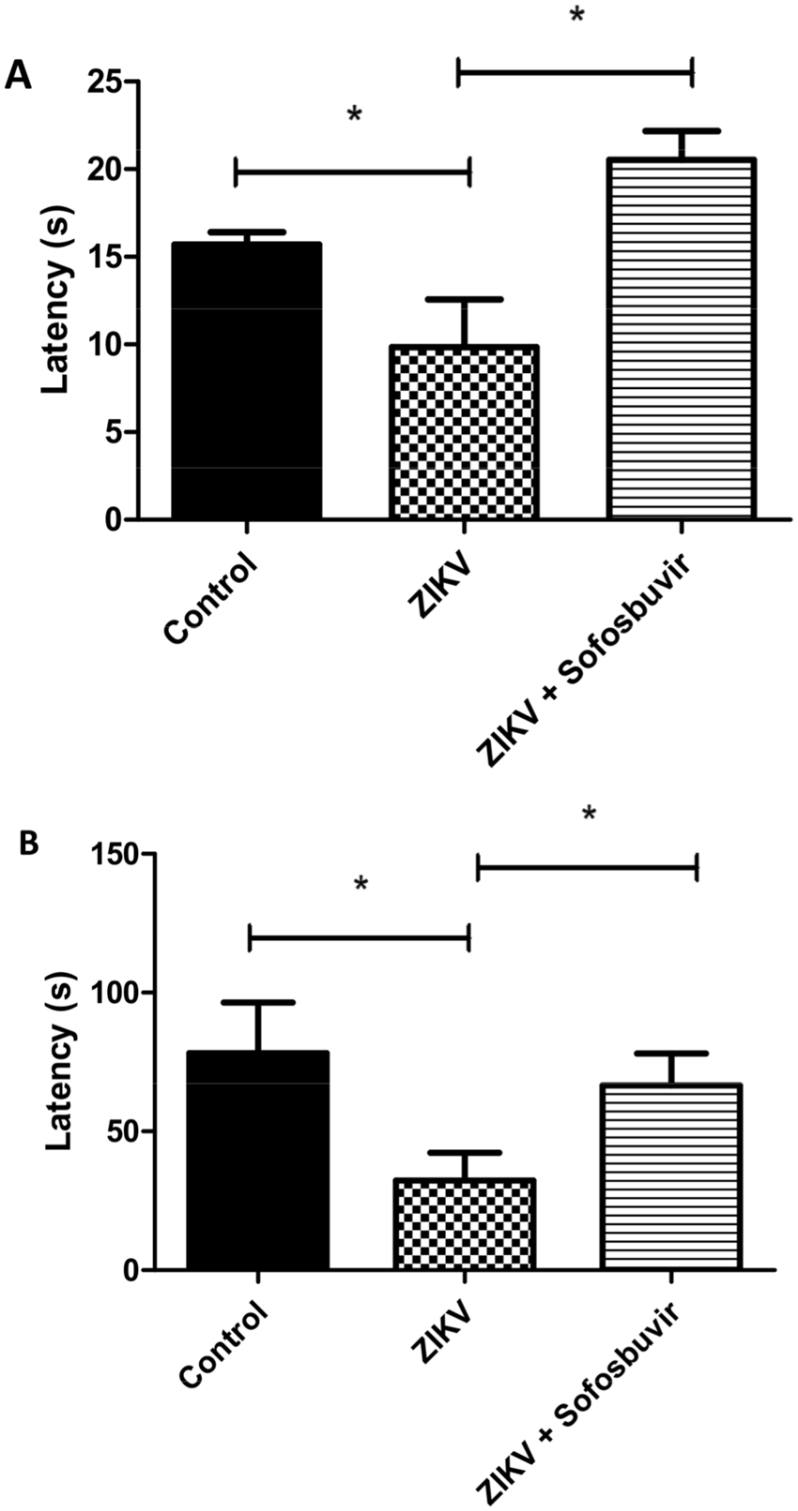
Sofosbuvir prevents memory loss in animals that survive ZIKV infection. Animals that survived from ZIKV﻿ neonatal infection, untreated or﻿ pretreated with sofosb﻿uvir, were kept and tested for behavioural sequelae in learning and memory after 60 days. The time to find the platform, according to MWM test, was assessed. Trial in the absence of the platform was conducted (**A**) (*P < 0.05, Student’s t test). In panel A, latency represents the time spent exploring the quadrant where the platform was located before removal, and the data are expressed as the means ± SEM (n = 5 to 8 per experimental group). Aversive memory was evaluated by freezing behaviour 24 h post-training session, whereas mice were allowed to explore the aversive environment during 180 s followed by two foot shocks (0.6 mA, 3 s) (**B**). In panel B, latency represents the time spent without movement (freezing) during 180 s, and the data are expressed as the means ± SEM (n = 5 to 8 per experimental group).

Moreover, some ZIKV-infected mice did not succumb to the infection (Fig. 1A). We kept convalescent mice from ﻿acute ZIKV infection for 6 to 8 weeks to further monitor behavioural sequelae. We applied the Morris water maze test to assess hippocampal learning and memory. On the learning tests, training to find a platform 2 cm beneath the surface of the water was carried out for 4 days. Healthy control animals and survivors from ZIKV infection responded similarly to learning, independently of whether infected animals were treated (Fig. S2). On the 5^th^ day after training, the platform was removed, and memory was evaluated by measuring the time to stay in the platform’s quadrant (latency). Untreated ZIKV-infected mice did not stay on the quadrant where the platform had been previously located in comparison to control (uninfected) and sofosbuvir-treated ZIKV-infected mice (Fig. 6A).

Subsequently, an amygdala-dependent aversive memory test was performed (freezing test). This test consists of two foot shocks on mice. On the next day, mice are exposed to the same environment without a shock, when latency is measured. Our data showed that untreated ZIKV-infected animals lost the aversive memory, whereas the sofosbuvir-treated ZIKV-infected mice and healthy controls behaved normally (Fig. 6B). Our results indicate that, in addition to the increase in survival by sofosbuvir, this drug also prevents ZIKV-induced behavioural sequelae at the levels of neuromotor impairment and memory loss.

## Discussion

In recent years, the risk perception on ZIKV infection has increased substantially. Although Zika fever is a mild and self-limiting disease in most cases^27^, ZIKV-associated morbidities have been described^2, 3^. Since 2013/2014, ZIKV has spread explosively across immunologically naïve populations throughout the world, especially in the Americas^28^. For instance, in Brazil during 2015, it is estimated that over 4 million people were affected by this virus^29^. Major concerns were raised due to the association of ZIKV infection with neurological disorders during foetal development and adulthood^2, 3^. More recently, Zika-associated deaths have also been reported^4, 5^. We and others have shown that sofosbuvir, a clinically approved drug against HCV, shows strong antiviral activity against ZIKV^12–14^. In particular, we showed that sofosbuvir is functionally active against ZIKV in cells derived from peripheral organs and the CNS by targeting viral RNA polymerase^13^. To advance the pre-clinical development of sofosbuvir as an anti-ZIKV drug, we further examined whether this uridine analogue is active *in vivo*.

Pharmacological studies on animal models with representative genetic heterogeneity, such as Swiss outbred mice, may allow further exploration of the generated data to a broader population^16^. The neonatal mouse model of ZIKV infection, at postnatal days 0 and 1, has been characterized to study viral physiopathology on the nervous system^18^. The authors considered that C57BL/6 and Swiss mice responded similarly to ZIKV^18^. We chose the outbred animal model because it may represent a more natural system. For logistic reasons, it was unfeasible to use the day 0 or 1 neonatal mouse model^18^. Under our conditions, it was necessary to have enough time to accommodate the animals after birth in the experimental laboratory and then pretreat 1 day before infection. Thus, we infected 3-day-old Swiss mice to monitor the benefits of sofosbuvir treatment on the survival of ZIKV-infected animals. Of note, we did not aim to compare the cellular and molecular aspects of postnatal neurodevelopment nor physiopathology of ZIKV infection with previous works^18^ because we used older neonatal mice. Older animals displayed limited susceptibility to ZIKV infection when compared to the work of van den Pol *et al*.^18^. Although we used a severe virus challenge throughout our investigation, a similar dose of virus has been used by a different route of inoculation (intracranially) to investigate the physiopathology of ZIKV infection in the brain^23^.

Treatments with sofosbuvir were carried out at 20 mg/kg/day because this dose was administered to mice during preclinical development. The studies using this dose supported the clinical dossier to approve the safe and effective use of sofosbuvir in humans at 400 mg/day to treat HCV infection^25^. Thus, as a proof of concept, our findings consistently show that sofosbuvir, at a pragmatic concentration, possesses antiviral activity robust enough to provide protection against severe doses of virus. Under our conditions, less than 5% of all the animals assayed survived. Sofosbuvir increased the time of survival and reduced the mortality of infected animals. We also identified an associated likelihood of decreased mortality when comparing pretreatment with late initiation of treatment (2 days after infection). These data may provide two important general notions: i) earlier sofosbuvir administration to ZIKV-infected mice leads to better antiviral results and reduced mortality; and ii) regardless of timing, it is more opportune to administer sofosbuvir rather than leave the animals without treatment for the sake of survival. This narrow and early time frame for antiviral intervention is common to other acute viral infections^30^. Mortality to pandemic influenza, for example, is reduced if neuraminidase inhibitors are administered early in the time course of infection, preferentially within 2.5 days of infection^30^. Sofosbuvir-treated animals had reduced mortality risk and also lived longer.

Moreover, sofosbuvir pretreatment prevented ZIKV mortality, suggesting its potential use for pre-exposure prophylaxis. We envision that prophylactic activity is a distinguishable feature of sofosbuvir against Zika. If safe, prophylactic drugs could be used by individuals at high risk of complications by a given infectious agent. Pregnant women and their babies are at the highest risk of ZIKV-associated morbidities. Sofosbuvir has a record of safety, even when the indiscriminate use of this substance occurred in limited numbers of pregnant women^31^. In addition, more accurate clinical-based evidence may start to emerge in late 2018 because a clinical trial of sofosbuvir on pregnant women is ongoing^32^.

ZIKV detection in the kidney, urine, plasma, and spleen before reaching the brain^33–35^ has been associated as a hallmark of the natural history of ZIKV infection. Sofosbuvir effects on survival were associated with an inhibition of acute virus infection and spread through different anatomical compartments. Since sofosbuvir reduced acute virus loads, sofosbuvir-treated ZIKV-infected animals displayed fewer foci of brain microhaemorrhage when compared to their untreated counterparts. Moreover, sofosbuvir-treated mice responded properly to neuromotor reflexes (righting), whereas untreated ZIKV-infected animals had severe impairment of this parameter.

In the long-term analysis of animals that survived, we noticed that ZIKV-infected animals had behavioural sequelae compatible with memory impairment. This is consistent with virus-induced cell death and cerebral inflammation in memory-forming areas^14, 18, 23, 36, 37^. Sofosbuvir-treated ZIKV-infected mice survived longer and in more numbers than untreated animals. The surviving animals were also healthier, responding to memory testing behaviour consistent with uninfected control mice.

Altogether, our results indicate that sofosbuvir treatment at a pharmacologically relevant concentration inhibits ZIKV replication *in vivo*, reducing mortality and blocking behavioural sequelae in the short- and long-term analysis. These results are an important proof of concept and are consistent with a prospective necessity of antiviral drugs to treat ZIKV-infected individuals.

## Electronic supplementary material

Figure S1 and S2

Supplementary Video
