# Supplementary material for: Sofosbuvir protects Zika virus-infected mice from mortality, preventing short- and long-term sequelae
Source: Sci Rep. 2017 Aug 25;7:9409. doi: 10.1038/s41598-017-09797-8 (PMC5573375; doi:10.1038/s41598-017-09797-8)
Supplement: Supplementary file 1 — Figure S1 and S2 [file 41598_2017_9797_MOESM1_ESM.pdf]

Supplementary information (online only)

Sofosbuvir protects Zika virus-infected mice from mortality, preventing short- and long-term sequelae

André C. Ferreira[1#], Camila Zaverucha-do-Valle[1#], Patrícia A. Reis[1#], Giselle Barbosa-Lima[2], Yasmine Rangel Vieira[2], Mayara Mattos[1], Priscila de Paiva Silva[1], Carolina Sacramento[1], Hugo C. de Castro Faria Neto[1], Loraine Campanati[3], Amilcar Tanuri[4], Karin Brüning[5], Fernando A. Bozza[1,2], Patrícia T. Bozza[1], and Thiago Moreno L. Souza[1,2,6]*

[1]Laboratório de Imunofarmacologia, Instituto Oswaldo Cruz (IOC), Fundação Oswaldo Cruz (Fiocruz), Rio de Janeiro, RJ, Brazil.

[2]Instituto Nacional de Infectologia (INI), Fiocruz, Rio de Janeiro, RJ, Brazil.

[3]Instituto de Ciências Biomédicas, Universidade Federal do Rio de Janeiro, Rio de Janeiro, RJ, Brazil.

[4]Instituto de Biologia, Universidade Federal do Rio de Janeiro (UFRJ), Rio de Janeiro, RJ, Brazil.

[5]BMK Consortium: Blanver Farmoquímica Ltda; Microbiológica Química e FarmacêuticaLtda; Karin Bruning & Cia. Ltda, Brazil

[6]National Institute for Science and Technology on Innovation on Neglected Diseases (INCT/IDN), Center for Technological Development in Health (CDTS), Fiocruz, Rio de Janeiro, RJ, Brazil.

[#]These authors contributed equally to this work

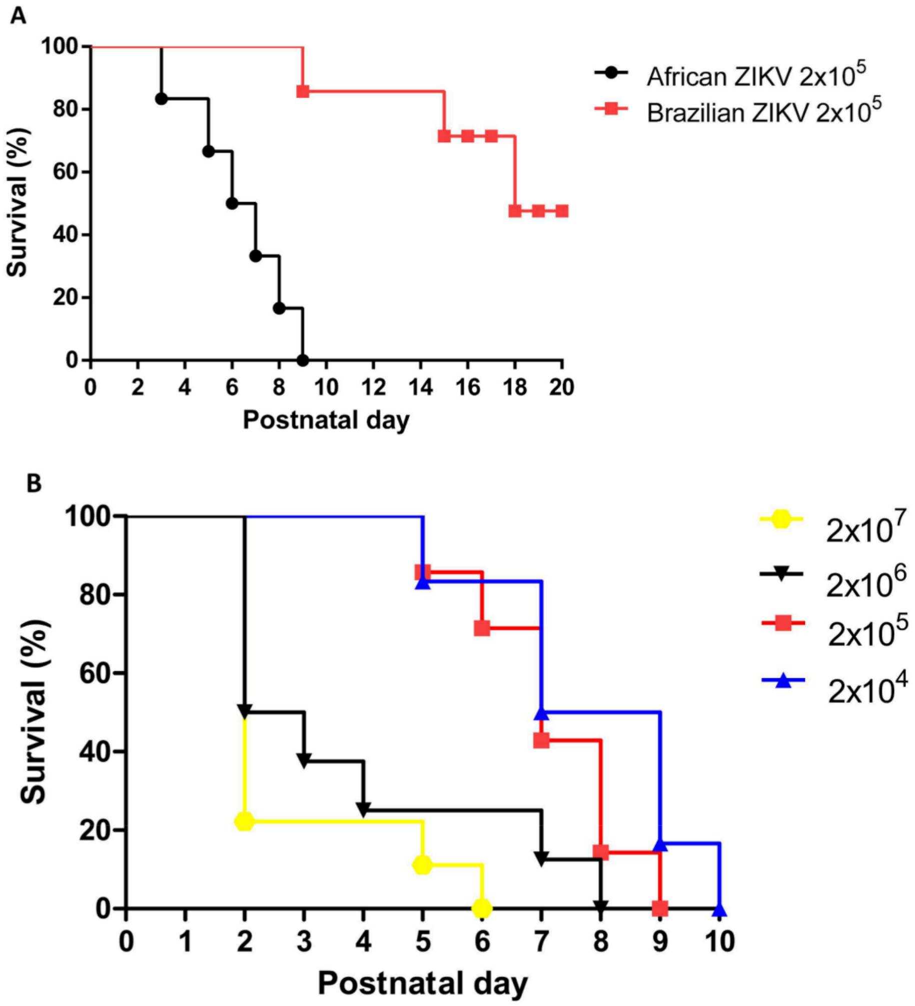

Figure S1. Survival curves of ZIKV-infected mice. Three-day-old Swiss mice were infected with African or Brazilian ZIKV strains with the indicated PFUs (A). Alternatively, the indicated PFUs of African ZIKV were used to infect the mice (B). Survival was assessed daily, up to 20 days. Independent experiments were performed with 7 animals per group (n = 21).

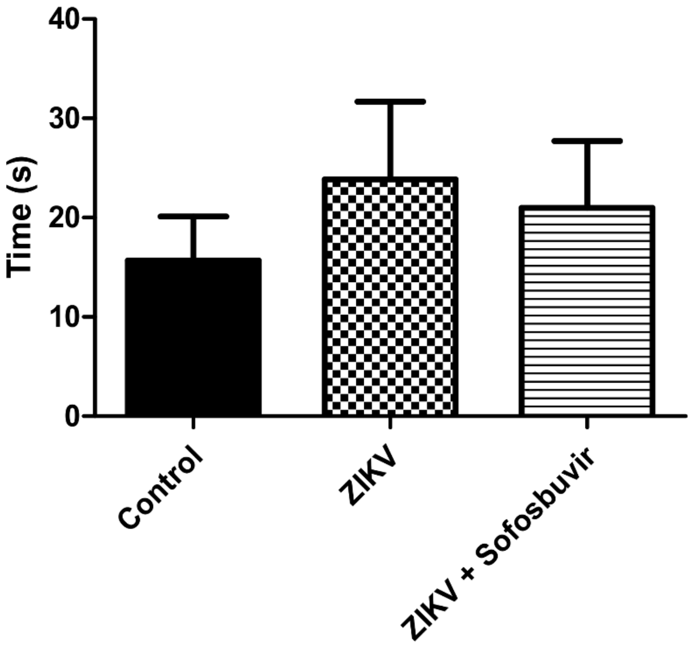

Figure S2. ZIKV effect on the long-term learning of mice. Three-day-old Swiss mice were infected with ZIKV (2 x 10^7 PFU) and treated with sofosbuvir (SF) or not (nil) beginning at 1 day before infection. Animals that survived were kept and tested for behavioural sequelae in learning after 60 days. The time to find the platform, according to the MWM test, was assessed. The data are expressed as the means ± SEM (n = 6 to 11 animals per experimental group).
